# Single-cell transcriptomic landscape and the microenvironment of normal adjacent tissues in hypopharyngeal carcinoma

**DOI:** 10.1186/s12864-024-10321-2

**Published:** 2024-05-17

**Authors:** Rui Guan, Ce Li, Fangmeng Gu, Wenming Li, Dongmin Wei, Shengda Cao, Fen Chang, Dapeng Lei

**Affiliations:** 1 Department of Otorhinolaryngology, NHC Key Laboratory of Otorhinolaryngology (Shandong University), Qilu Hospital, Cheeloo College of Medicine, Shandong University, 107 West Wenhua Road, Shandong, 250012 China; 2 Cheeloo College of Medicine, Shandong University, Jinan , Shandong, 250012 China

**Keywords:** Human hypopharyngeal carcinoma, normal adjacent tissues, Single-cell RNA sequencing, Epithelial cells, Fibroblasts

## Abstract

**Background:**

The cellular origin of hypopharyngeal diseases is crucial for further diagnosis and treatment, and the microenvironment in tissues may also be associated with specific cell types at the same time. Normal adjacent tissues (NATs) of hypopharyngeal carcinoma differ from non-tumor-bearing tissues, and can influenced by the tumor. However, the heterogeneity in kinds of disease samples remains little known, and the transcriptomic profile about biological information associated with disease occurrence and clinical outcome contained in it has yet to be fully evaluated. For these reasons, we should quickly investigate the taxonomic and transcriptomic information of NATs in human hypopharynx.

**Results:**

Single-cell suspensions of normal adjacent tissues (NATs) of hypopharyngeal carcinoma were obtained and single-cell RNA sequencing (scRNA-seq) was performed. We present scRNA-seq data from 39,315 high-quality cells in the hypopharyngeal from five human donors, nine clusters of normal adjacent human hypopharyngeal cells were presented, including epithelial cells, endothelial cells (ECs), mononuclear phagocyte system cells (MPs), fibroblasts, T cells, plasma cells, B cells, mural cells and mast cells. Nonimmune components in the microenvironment, including epithelial cells, endothelial cells, fibroblasts and the subpopulations of them were performed.

**Conclusions:**

Our data provide a solid basis for the study of single-cell landscape in human normal adjacent hypopharyngeal tissues biology and related diseases.

**Supplementary Information:**

The online version contains supplementary material available at 10.1186/s12864-024-10321-2.

## Background

The hypopharynx, otherwise referred to as the laryngopharynx, is the lowest part of the three pharyngeal divisions. It is located between the upper perineal margin plane and the lower annular cartilage plane, and connects downward into the esophagus. Anatomically, the hypopharynx is usually defined as the point at which the anterior part of the pharynx divides into the larynx, followed by the posterior pharyngeal wall, the piriform sinus, and the posterior annular region leading to the entrance of the esophagus [1]. As part of the pharynx, the hypopharynx plays a primary physiologic function as a cavity through which food and water pass through by the act of swallowing [2]. The hypopharynx also has key roles in breathing, speech formation [3], defense protection and partial immune functions. As these are key functions required for daily life, it is important to ensure a deep understanding of the hypopharynx functional pathways and how they may be affected by external factors. This understanding will also guide the optimal treatment protocols for diseases related to the hypopharynx. As the cavity that connects the external and internal environments, the hypopharynx is susceptible to different harmful factors such as alcohol [4], smoke, viral infection [5] and medicine [6], resulting in many different diseases within the hypopharynx. These include pharyngitis [7], laryngopharyngeal reflux [8], pharyngeal emergencies [9] and hypopharyngeal cancers, which are usually squamous cell carcinoma (SCC) and have the worst prognosis among head and neck cancers, with 5-year survival rates as low as 29% [10].

Normal adjacent tissues (NATs), also known as non-tumor tissues, are usually considered as a control group in tumor-related studies. A comprehensive analysis of transcriptomes and genome-derived haplotype-specific somatic copy number alterations suggested that the NAT is a unique intermediate state between healthy tissue and tumor and may accumulate oncogenic events [11]. In the process of diagnosis and treatment of tumors, cancer is usually treated after the formation process, which leads to the exploration of early cancer screening indicators and methods has become a problem. At present, there are related studies on paracancerous tissues of a variety of cancers, including liver cancer [12], breast cancer [13], urinary system tumors [14], etc. It is found that the adjacent tissues have the characteristics of early occurrence of cancer, and the molecular and biochemical characteristics of adjacent tissues are also related to the malignant degree of tumors and the prognosis of patients. Paracancerous normal tissue-derived organoids exhibit partial tumor-like characteristics at the transcriptome level, but retain normal genomic and global DNA methylome characteristics [15]. So we thought that normal tissues adjacent to cancer could be used to study some molecular changes that may occur early in cancer development.

Recent advances in single-cell genomics have provided avenues to explore genetic and functional heterogeneity at cellular resolution [16, 17]. Single-cell sequencing technology is a new technology for high-throughput sequencing of genomes, transcriptomes and epigenetic groups at the single cell level [18]. In similar cell types, gene expression may be heterogeneous. The use of conventional transcriptome sequencing technology would yield the average expression levels of genes within a tissue [19], masking differences between individual cells, when the pathogenesis of disease may only be related to one cell type [20]. RNA sequencing provides a powerful approach to characterize the clonal diversity of tumor cells and explore the role of atypical cells in tumor development. There is a growing belief among scientists that single-cell RNA sequencing can uncover the heterogeneity of head and neck malignancies brought about by changes in hypoxia, stress, epithelial differentiation [21] and metabolism [22]. This could promote further understanding of the ability of different cell types to invade and metastasize [23–25], and how the surrounding microenvironments influence these processes. Previous studies have reported single-cell transcriptome analysis of primary and metastatic tumor ecosystems in head and neck cancer, particularly oral head and neck squamous cell carcinoma (HNSCC) tumors [25]. Several studies have also focused on the single-cell components and immune microenvironment of hypopharyngeal carcinoma [26, 27]. Nonetheless, there are no reports at the single-cell transcriptome level for normal adjacent tissues to hypopharyngeal carcinoma.

Tumor microenvironment (TME), is a complex and comprehensive system mainly composed of tumor cells, surrounding immune and inflammatory cells, tumor-associated fibroblasts, and nearby interstitial tissue, microvessels, as well as various cytokines and chemokines [28]. It can be divided into immune microenvironment dominated by immune cells and non-immune microenvironment dominated by fibroblasts. TME cells and their secreted molecules are now recognized to play a key role in the pathogenesis of cancer and are therefore attractive therapeutic targets [29]. Depending on the organ in which the tumor arises, and the patient characteristics, the cellular composition and functional status of the TME will vary. Fibroblasts and endothelial cells were significantly enriched in our single-cell detection of paracancerous tissues. The microenvironment surrounding the tumor is essential for understanding recurrence and in developing surgical strategies [30]. Considering the response and characterization of tumor components and subtypes in paracancerous tissues, we believe that the study of fibroblasts and endothelial cells in paracancerous tissues is of great significance.

It is generally accepted that lymphocytes are the smallest white blood cells and can be divided into T lymphocytes, B lymphocytes and natural killer (NK) cells based on their migration, surface molecules and functions. In view of the different characteristics, bone marrow-derived B cells are mainly involved in humoral immunity, while thymus-derived T cells are involved in cellular immunity. As the main component of lymphocytes, T cells have a variety of biological functions, such as direct killing of target cells, assisting or inhibiting B cells to produce antibodies, and responding to specific antigens and mitotropins [31, 32], and producing cytokines, etc. T cells are activated by antigen-specific signals and ‘co-stimulatory’ signals [33]. T cells confer cellular immunity whereby they specifically bind target cells to directly kill those target cells, and by releasing lymphokines, which are important in expanding and enhancing the immune effect [34, 35]. The main T cell cluster was divided into four subclusters, which were annotated as regulatory T cells (Tregs), conventional CD4 T helper cells (CD4 T +  + CONV), and two cytotoxic CD8 T cell populations [25]. They proposed that the different T cell expression states may be important in understanding and predicting responses to checkpoint immunotherapies [36]. Based on the unique physiological function of T cells and the distinctive advantage of single-cell sequencing, we postulated that single-cell sequencing can assist in expanding on current understanding of the typical tissue structure and function and the interactions between different cells, in particular the role that T cells play in normal tissue and disease progression.

In this study, we were interested to gain new insights on the common and specific characteristics of different cell types within normal tissues of the hypopharynx as well as to understand the interaction between varied cell types of hypopharynx and immune cells. We prepared single-cell suspensions of human hypopharyngeal tissue and performed scRNA-seq using a high-throughput droplet mediated scRNA-seq platform. A total of 39,315 high-quality human cells were obtained from five donors (HSCC_N1, N2, N3, N4 and N5). The population of cells was made up of 10,723 epithelial cells, 2527 endothelial cells, 3987 mononuclear phagocytes, 884 T cells, 723 fibroblasts, 328 plasma cells, 270 B cells, 196 mural cells and 117 mast cells. RNA sequencing of this population of cells produced a single-cell transcriptome dataset. As different types of cells play important roles in the early stage of carcinoma, we grouped the cells based on different expression signatures and studied the differences between groups. Analysis of this substantial single-cell transcriptome data set enabled us to validate previously reported adjacent to tumor tissues-related susceptibility genes. More importantly, the adoption of unbiased cell classification allowed us to discover novel genes with specific expression in certain cell types. In summary, our obtained data provide richer transcriptome information for normal adjacent to hypopharyngeal cancer cells, and provide an important reference for the accurate classification of cells and the study of the relationship between different cells and diseases.

## Methods

We outline the hypopharyngeal scRNA-seq method. The whole process includes sample collection, tissue dissociation and preparation, single-cell RNA sequencing process, and scRNA-seq quantitative and statistical analysis.

### Sample collection, processing and transportation

Fresh human hypopharyngeal samples (normal and neoplastic) were obtained from patients undergoing surgery under sterile conditions at the Department of Otolaryngology, Qilu Hospital, Shandong University. After tissue dissection, the tissue of interest was cut into large bean-grain sized blocks (approximately 100 mg), washed twice with sterile phosphate buffer (PBS), and immediately treated with GEXSCOPE® tissue preservation solution (Singleron, Nanjing, China). Stored tissue samples were transported on ice to Singleron Biotechnologies.

### Tissue dissociation and preparation of single cell suspension

Samples were washed 3 times in Hank's Balanced Salt solution (HBSS) and then cut into 1–2 mm pieces. The Tissue blocks were then dissociated with 2 mL GEXSCOPE® Tissue Dissociation Solution (Singleron) for 15 min at 37 °C with continuous agitation. After digestion, samples were filtered through a 40-micron sterile filter followed by centrifugation at 200 g for 5 min. The supernatant was then discarded and the precipitate was re-suspended in 1 ml PBS (HyClone). To remove Red Blood cells, 2 mL GEXSCOPE® Red Blood Cell Lysis Buffer (Singleron) was added for 10 min at 25 °C. The combined solution was then centrifuged at 500 × g for 5 min and re-suspended in PBS. Samples were stained with trypan blue (Sigma) and evaluated by light microscopy.

### Single cell RNA sequencing

Single-cell suspensions of 1 × 10^5 cells /mL were prepared in PBS (HyClone). Single-cell suspensions were then loaded into the microfluidic device and scrNA-seq libraries were constructed according to the Singleron GEXSCOPE® Single-cell RNA Library Kit (Singleron Biotechnologies protocol) [37]. Individual libraries were diluted to 4 nM and pooled for sequencing. Pools were sequenced using 150 bp paired-end Reads on an Illumina HiSeq X.

### Quantification and statistical analysis of scRNA-seq

Using fastQC v0.11.4 (https://www.bioinformatics.babraham.ac.uk/projects/fastqc/) to deal with the raw Reads, to use the internal pipeline generated gene expression profile. In brief, we filtered readings without poly-T tails and extracted cell barcodes and unique molecular identifiers (UMI). Trim the adapter and poly-A tail used FASTP (version 1) [38]. The readouts were aligned with the human reference genome GRCh38 (ACC.no. Gca_000001405.15.) transcriptome using STAR (v2.6.1b) before using integrated version 92 gene annotations (FASTP 2.5.3A and featureCounts 1.6.2) [39]. The readouts were grouped with those of a human reference genome pair with the same cell barcode, UMI, and genes using integrated version 92 Gene Annotations (FASTP 2.5.3A and featureCounts 1.6.2) to calculate the number of UMIs per gene per cell. UMI counts for each cell barcode were used for further analysis. Cell type identification and cluster analysis were performed using the Seurat program (//satijalab.org/seurat/, R package, V.3.0.1) [40, 41]. Used the read.table function to read the UMI count table into R. Then set the parameter resolution of the FindClusters function to 0.6 to identify clusters. The FindMarkers function was used to identify differentially expressed genes (DEGs) between different samples or between consecutive clusters using the Seurat program. We used clusterProfiler software to perform GO functional enrichment analysis on gene sets to find biological functions or pathways significantly associated with differentially expressed genes [42]. Expression matrix files for subsequent analyses were generated based on gene numbers and UMI numbers (Table 1).

**Table 1:** Detailed QC of FASTQ files

Sample name	Sample ID	Estimated Number of Cells	Fraction Reads in Cells	Mean Reads per Cell	Median UMI per Cell	Total Genes	Median Genes per Cell	Sequencing Saturation	Sequencing depth
HSCC_N1	M-21240647	5,001	29.20%	78,507	3,627	26,855	1,370	65.39%	78,507
HSCC_N2	M-21240474	11,530	48.62%	35,422	5,625	30,694	1,754	46.93%	35,422
HSCC_N3	M-21240488	8,588	49.43%	35,601	5,990	30,421	1,999	46.72%	35,601
HSCC_N4	M-22023264	7,961	47.41%	55,992	5,552	30,263	1,804	52.03%	55,992
HSCC_N5	M-22023021	6,235	40.03%	62,622	4,959	29,362	1,707	63.03%	62,622

### Quality control, dimensionality reduction, clustering

Cells were filtered according to the following parameters: less than 200 or the top 2% of genes and the top 2% of UMI counts were excluded. Cells with more than 20% mitochondria were also depleted. After filtration, 39,315 cells were retained for downstream analysis, with an average of 1727 genes and 5151 UMI per cell. For reduction and clustering, we used the functions in Seurat V3.1.2 (Satijalab/Seurat:3.1.2). Gene expression was normalized and scaled using NormalizeData and ScaleData. Principal component analysis (PCA) FindVariableFeautres was used to select the top 2000 genes with variable expression. FindClusters divided the cells into 31 clusters using the first 20 principal components and a resolution parameter of 1.2. For subclustering of nine cell types, the resolution was set differently (0.2 in epithelial cells, 0.1 in fibroblasts, 0.8 in endothelial cells, 0.8 in mononuclear phagocytes, 0.8 in T cells. Uniform manifold approximation and projection (UMAP [43]) algorithm was applied to visualize cells in a two-dimensional space.

### Differentially expressed gene (DEG) analysis

Seurat FindMarkers selects genes as DEGs that are expressed in ≥ 10% of cells within the cluster with average log twofold change greater than 0.5 based on Wilcoxon likelihood ratio tests using default parameters.

### Cell cycle status evaluation

Scran package was used for G1 phase cell identification. Seurat package was used for G2 and M phase cell identification.

### Cell type annotation

The cell-type identity of each cluster was determined based on the expression of canonical markers found in DEGs, combined with knowledge from the literature.

### Trajectory analysis

To map cell subtype differentiation/transformation in different subtypes, we performed a pseudo-time trajectory analysis using Monocle2 [44]. To construct trajectories, differentially expressed genes were used to classify cells in order of spatial and temporal differentiation. DDRTree is used to perform FindVairableFeatures and dimensionality reduction. Visualize trajectories by plotting cell trajectories [45].

### Patient survival analysis

We analyzed the association between fibroblast subset marker genes and survival in patients with head and neck squamous cell carcinoma on GEPIA [46].

## Results

**Fig. 1:**
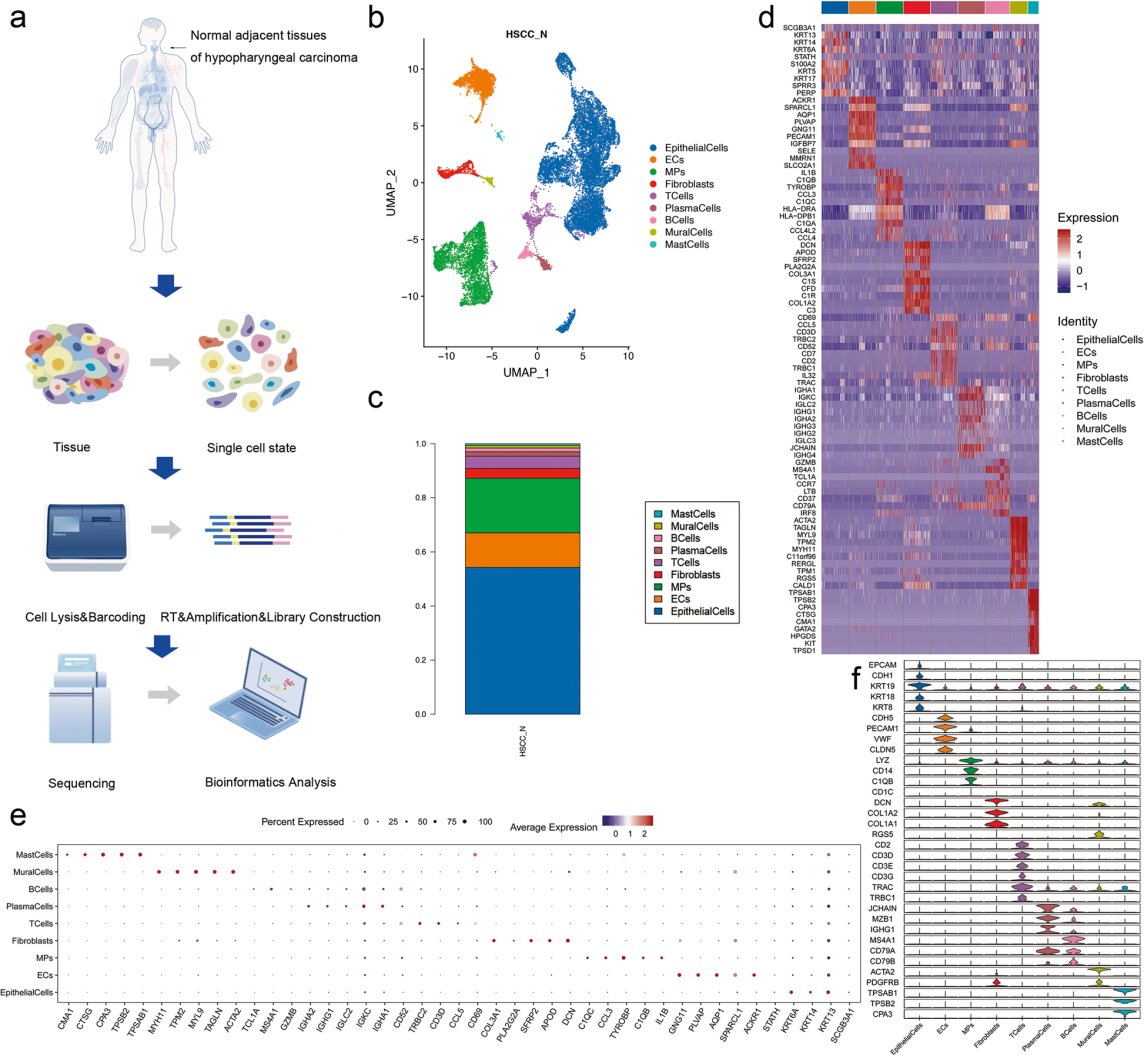
scRNA-seq reveals the cell population of the normal adjacent tissues (NATs) in hypopharyngeal carcinoma. (**a**) Overview of the scRNA-seq process using human hypopharynx normal adjacent tissue samples. (**b**) Uniform manifold approximation and projection (UMAP) plot showing the unbiased classification of adjacent cancer cells. (**c**) Bar chart showing the proportion of each cell type in each sample. (**d**) Heat map showing the top 10 marker genes of each cluster. (**e**) Bubble chart showing top five genes expressed in each cluster. (**f**) Violin plots representing the expression of marker genes in different clusters

Hypopharyngeal specimens from organ donors (five males) aged 41 to 74 years were freshly collected, dissected and digested into single cells (Supplementary Tables S1, S2, “Methods”). After collection, dissociation, and preparation of normal and pathological tissues, single cells were sequenced, quantified, and statistically analyzed (Fig. 1a). After QC, 39,315 high-quality adjacent-to-tumor hypopharyngeal cells were further analyzed (Table 2). We used the UMAP method to visualize cell clustering (Fig. 1b). Nine cell clusters were identified, with the number of cells in each cluster ranging from 117 to 10,723 cells (Fig. 1c). Based on the marker genes, we classified cells into clusters 1–9 corresponding to epithelial cells, endothelial cells (EC), mononuclear phagocytic system cells (MP), fibroblasts, T cells, plasma cells, B cells, parietal cells, and mast cells, respectively (Fig. 1d). In order to better determine the characteristics and functions of each of the nine subgroups, we analyzed the top five genes expressed in each cluster (Fig. 1e). We also tested the expression of the marker genes in each subset in other subsets to verify the specificity of the marker genes (Fig. 1f).

**Table 2:** Sequencing statistics based on cells

Sample name	Sample ID	Valid Reads	Q30 Bases in Barcode	Q30 Bases in UMI	Reads mapped uniquely to Genome	Base Pairs Mapped to Exonic Regions
HSCC_N1	M-21240647	91.98%	95.87%	94.73%	81.21%	83.81%
HSCC_N2	M-21240474	93.04%	95.99%	95.02%	87.23%	94.39%
HSCC_N3	M-21240488	92.95%	95.72%	94.59%	87.30%	93.13%
HSCC_N4	M-22023264	88.44%	95.49%	94.03%	63.65%	93.74%
HSCC_N5	M-22023021	93.18%	95.21%	93.55%	82.72%	88.51%

Mentioning single cell sequencing, the quality control (QC) is an unavoidable problem, as it is both related to the feasibility of the experiment and the reliability of the results. Seurat was used to ensure quality control of the scRNA sequencing output and to calculate the number of genes per cell, UMI number, and percentage of mitochondrial genes (Fig. S1a). Then we comprehensively analyzed the presentation of these three different data in all samples, on which the cells can be divided into different subtypes in a visual way (Fig. S1b). We also assessed the cell-cycle status of each cell (Fig. S1c) and calculated the proportion of cells in different cycle states for each subpopulation (Fig. S1d). Five samples were compared to remove batch effects (Fig. S1e). Because the proportion of mitochondrial genes reflects the state of the cell, the exclusion criteria were controversial. In this study, we selected cells that were conserved and filtered out with a percentage of mitochondrial genes > 20%.

**Fig. 2:**
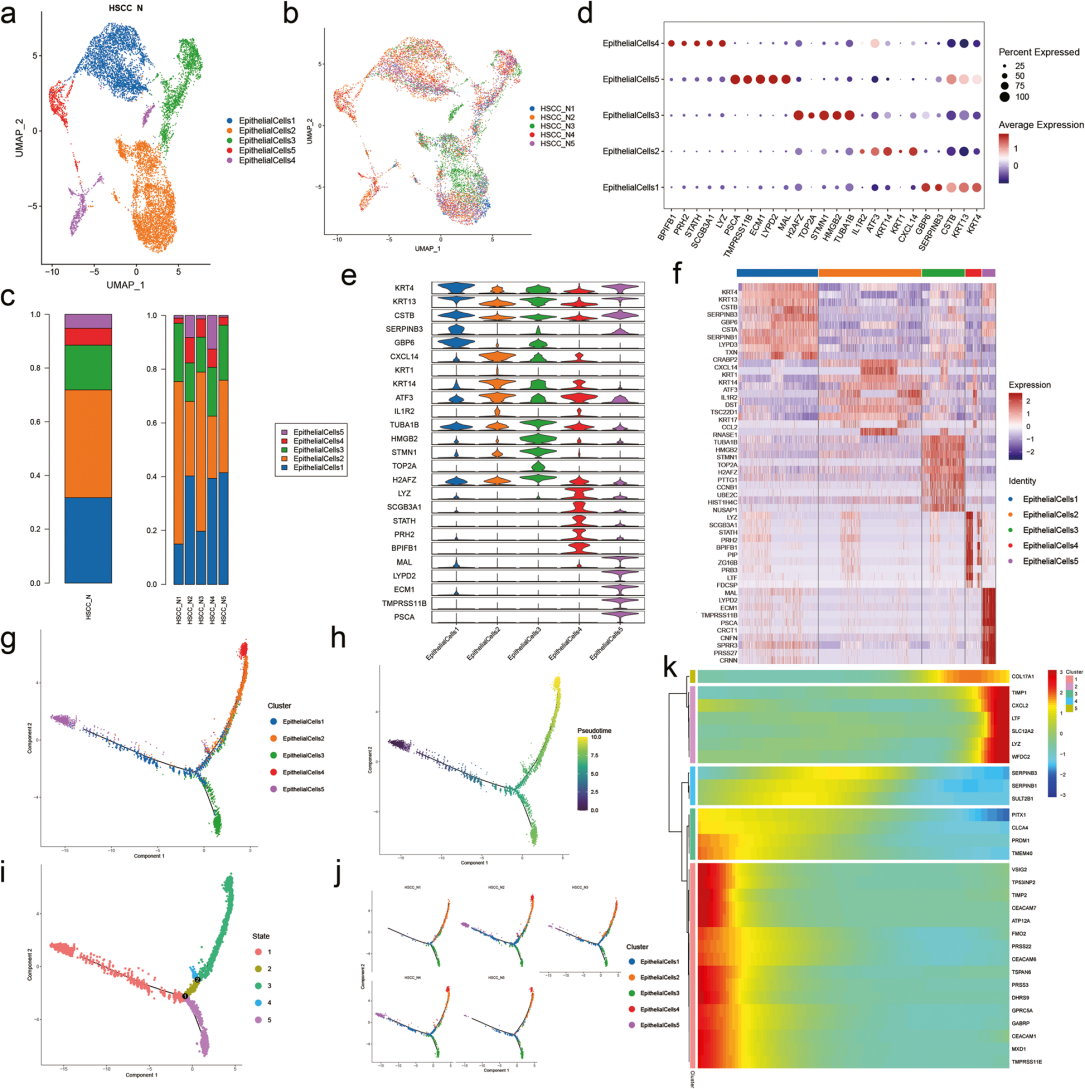
Subpopulations and pseudotime trajectory of epithelial cells. (**a**) Uniform manifold approximation and projection (UMAP) plot showing the sub classification of epithelial cells. (**b**) UMAP plot of five subtypes colored by samples. (**c**) Bar charts showing the proportion of each epithelial cells subtype totally and in each sample. (**d**) Violin plots representing the expression situation of common marker genes in different subtypes of epithelial cells. (**e**) Bubble chart showing five typical genes expressed in each subtype. (**f**) Heat map showing the top ten marker genes of each subpopulation. (**g**) Pseudotemporal trajectory of five epithelial cells types. (**h**) Pseudotime was coloured in a gradient from dark to light yellow, and the start of pseudotime is dark. (**i**) The pseudotime trajectory was divided into five different states. (**j**) The trajectory showing the distribution of cells from five samples. (**k**) Heat map for clustering the top genes that affected cell fate decisions

From the analysis, the detected cells were mainly divided into two basic cell types: non-immune cells and immune cells. The non-immune cells were very abundant, included epithelial cells, endothelial cells, fibroblasts and mural cells, which were also the focus of our study. Meanwhile, the immune cells also formed an integral part of the tissue microenvironment and included MPs, T cells, B cells, plasma cells and mast cells. Among all the nine cell types, epithelial cells were the most abundant with a total number of 10,723, almost one-third of the total cell number. In our analysis, epithelial cells were classified into five subpopulations (Fig. 2a). The presence of these unknown subpopulations was not deemed to be an artifact as they were detected in every sample (Fig. 2b). We counted the proportion of each epithelial cell subtype in each sample (Fig. 2c). In addition to this, we present a method for detailed classification of cell subsets by expression of canonical marker genes in epithelial cell subtypes (Fig. 2d) and chose top five typical genes expressed in each subtype (Fig. 2e). The top ten expressed genes can help us to understand the specificity of each subset of cells, and may have a crucial inspiration for the search of some genes worth exploring (Fig. 2f). Furthermore, We performed pseudo-time trajectories on all epithelial cells and showed fate decisions among them (Fig. 2g–k).

**Fig. 3:**
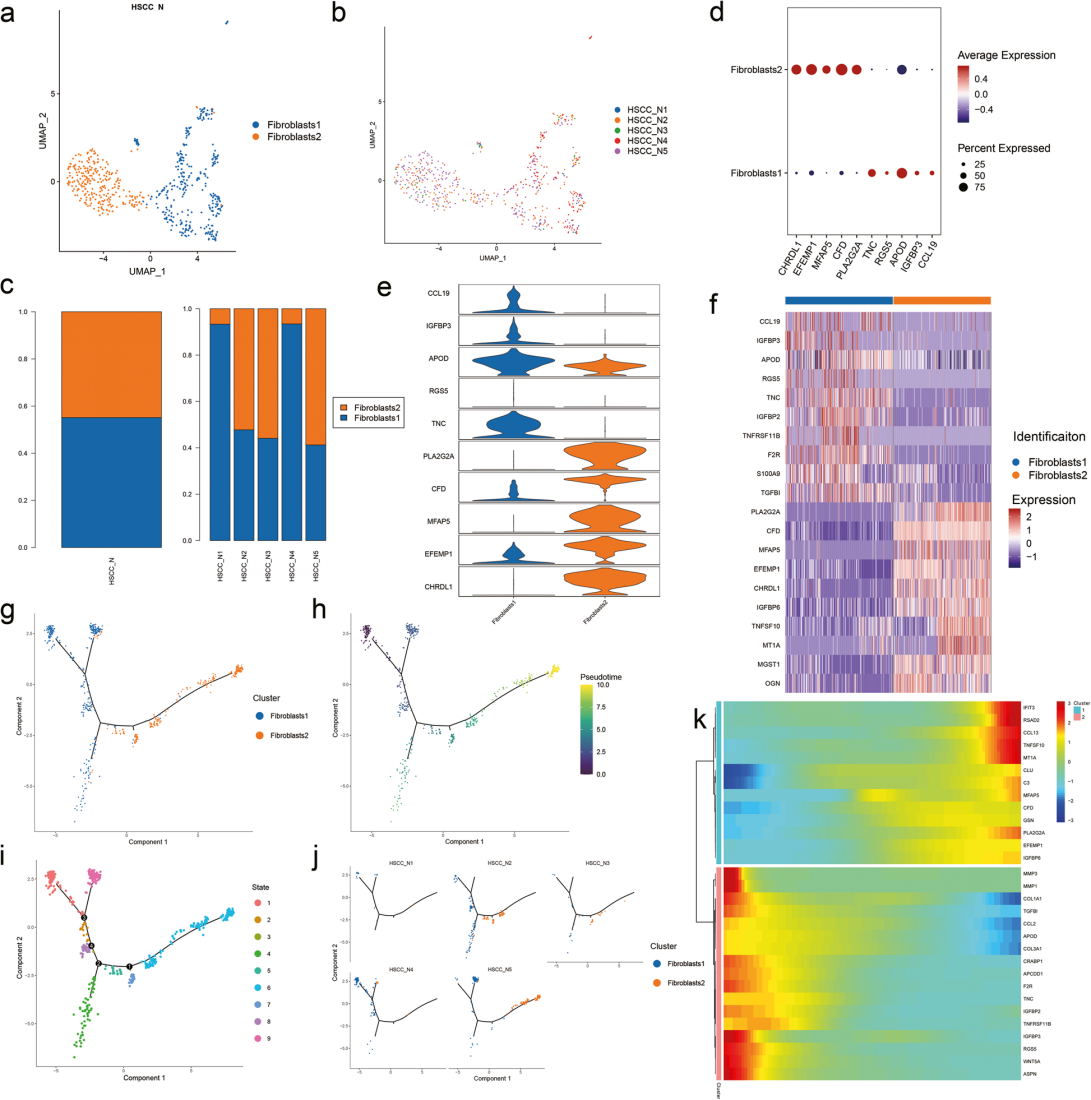
Subpopulations and pseudotime trajectory of fibroblasts. (**a**) Uniform manifold approximation and projection (UMAP) plot showing the sub classification of fibroblasts. (**b**) UMAP plot of five subtypes colored by samples. (**c**) Bar charts showing the proportion of each fibroblasts subtype totally and in each sample. (**d**) Violin plots representing the expression situation of common marker genes in different subtypes of fibroblasts. (**e**) Bubble chart showing five typical genes expressed in each subtype. (**f**) Heat map showing the top ten marker genes of each subpopulation. (**g**) Pseudotemporal trajectory of five fibroblasts types. (**h**) Pseudotime was coloured in a gradient from dark to light yellow, and the start of pseudotime is dark. (**i**) The pseudotime trajectory was divided into nine different states. (**j**) The trajectory showing the distribution of cells from five samples. (**k**) Heat map for clustering the top genes that affected cell fate decisions

Considering the important role of cancer associated fibroblasts (CAFs) in the tumor microenvironment, we also analyzed the specific types of fibroblasts in the adjacent tissues. In our analysis, fibroblasts were classified into two subpopulations (Fig. 3a). Fibroblasts of the five samples could similarly be roughly divided into two populations (Fig. 3b). Therefore, we counted the proportion of each fibroblasts subtype in each sample (Fig. 3c). In addition, we analyzed the expression of signature genes in these two positional subsets (Fig. 3d, e). The top ten expressed genes can help us to understand the specificity of the two subset of cells (Fig. 3f). The pseudo-time trajectories showed fate decisions among two types of fibroblasts. Pseudo-time trajectories could identify the origin and end points of differentiation according to the trajectory distribution of cell types and the expression changes of signature genes, and we noticed that the two subsets were mainly distributed at opposite ends of the time axis (Fig. 3 g). We thought cluster Fibroblast2 may represent a more advanced tendency to differentiate into tumor stages. After that, we performed pseudo-temporal sorting for all cells (Fig. 3 h), and found that all the cells could be divided into nine clusters according to four key time points (Fig. 3i). We then examined the distribution of each sample on the proposed time trajectories and found significant differences in cellular composition and differentiation estimates among samples (Fig. 3j). Then we presented the expression changes of marker genes during the course of pseudotime development (Fig. 3k).

**Fig. 4:**
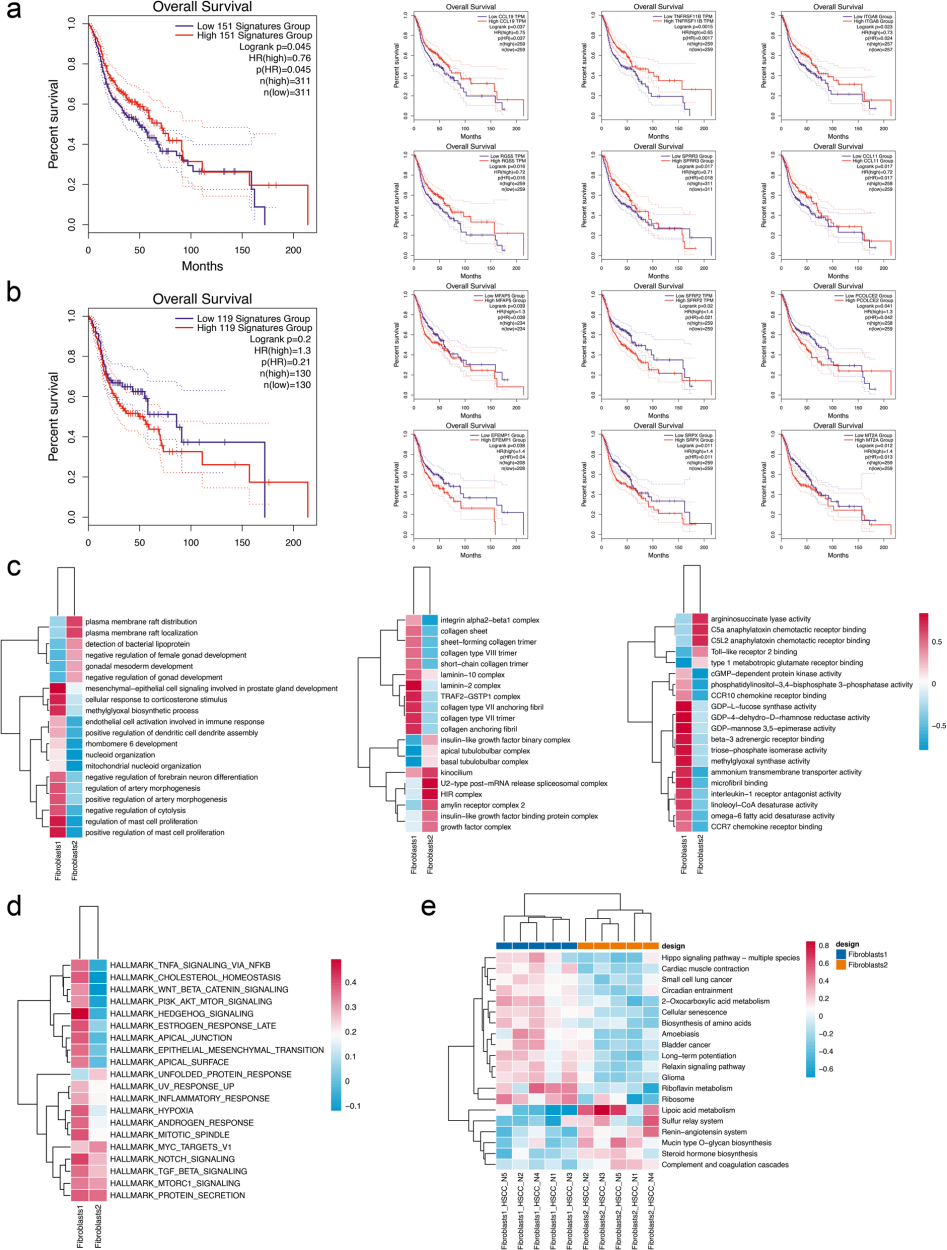
Survival analysis of fibroblast marker genes and related pathway analysis. (**a**) Survival analysis of fibroblasts1 marker genes in HNSCC patients. (**b**) Survival analysis of fibroblasts2 marker genes in HNSCC patients. (**c**) GO analysis of the two subtypes of fibroblasts. (**d**) Heatmap representing the top hallmarkers of two subtypes. (**e**) Heatmap showing KEGG pathways in two subtypes of five samples

Due to the different positions of the two subgroups in the stage of differentiation, we performed further queries on the signature genes of the two subgroups. We investigated the relationship between the expression of marker genes in two clusters and the survival of HNSC patients. We first selected the top 151 genes highly expressed in Fibroblasts1 (avg_logFC > 0.5) and analyzed their relationship with the survival of HNSCC patients. We found that high expression of these signatures was significantly associated with improved patient survival. The top 40 genes were further analyzed and nine genes were found to be significantly correlated with survival. Among them, eight genes showed their high expression was associated with improved survival, including CCL9, RGS5, TNFRSF11B, SPRR3 (cutoff-high 40%, cutoff-low 60%), ITGA8, CCL11, KRT13 and FMOD (Fig. 4a). We also selected the top 119 genes highly expressed in Fibroblasts2 (avg_logFC > 0.5) and made associated survival analysis with HNSCC patients. We found that high expression of these signatures was also connected with poor prognosis (HR value > 1). We searched top 40 genes in Fibroblasts2 and found six genes were found to be significantly correlated with survival. All of the upregulation of the six signatures was associated with poor survival, including MFAP5 (cutoff-high 55%, cutoff-low 45%), EFEMP1 (cutoff-high 60%, cutoff-low 40%), SFRP2, SRPX, PCOLCE2 and MT2A (Fig. 4b). We then explored the molecular functions and pathways involved in the two subpopulations. We first analyzed the gene ontology and found that cell components like insulin-like growth factor binding protein complex and growth factor complex, Toll-like receptor 2 binding and other biological process were enriched in fibroblasts2, which means this subtype related with cell growth, differentiation and the process of immune regulation (Fig. 4c). Hallmark pathway analysis helped us find the biochemical processes and roles that fibroblasts may participate in the transformation from normal microenvironment to tumor microenvironment, for instance, from Hedgehog signaling in fibroblasts1 to Myc signaling in fibroblasts2 (Fig. 4d). We also made KEGG analysis and showed heterogeneity across patients (Fig. 4e).

**Fig. 5:**
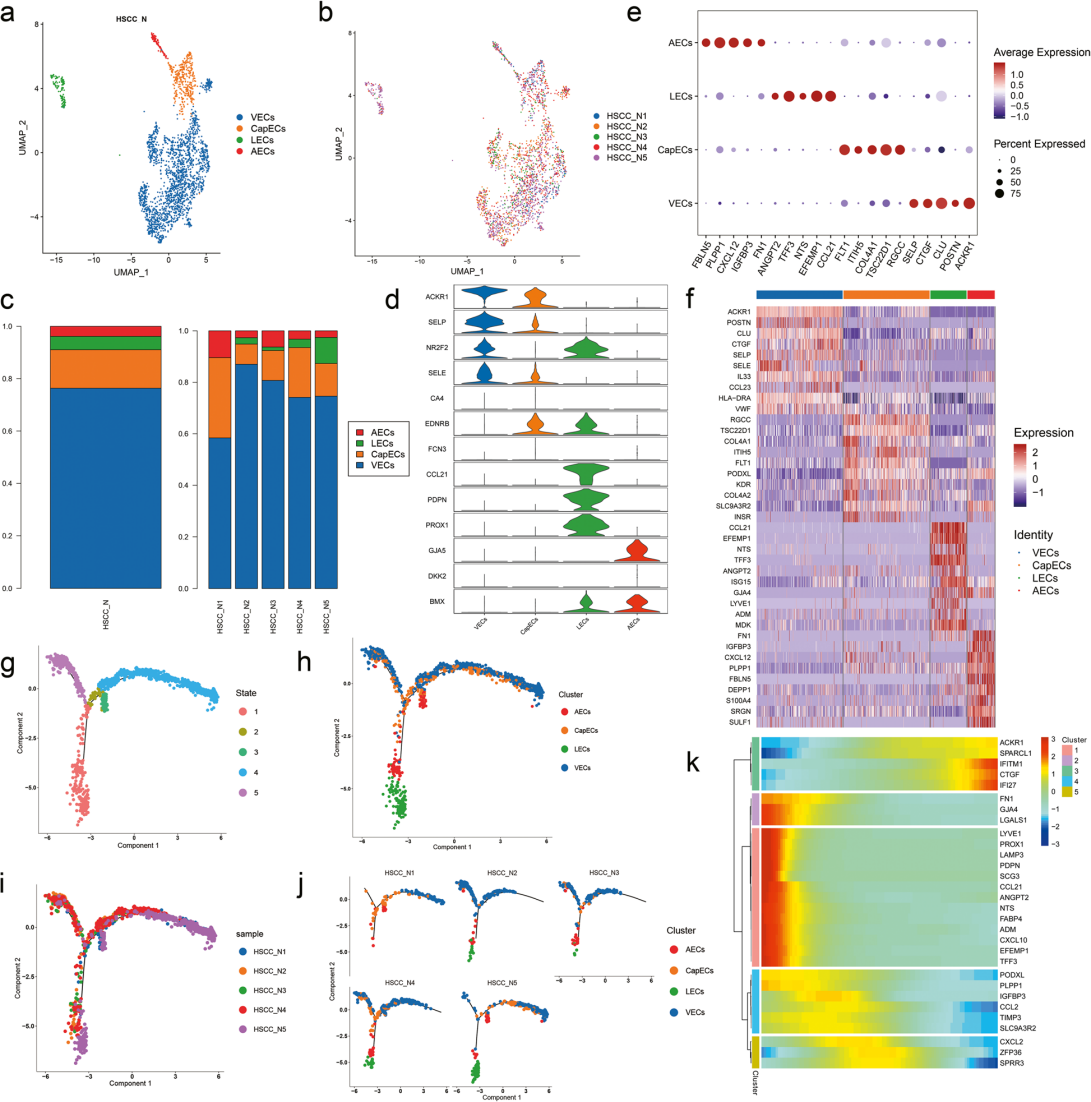
Subpopulations and pseudotime trajectory of endothelial cells. (**a**) Uniform manifold approximation and projection (UMAP) plot showing the sub classification of endothelial cells. (**b**) UMAP plot of cell distribution colored by samples. (**c**) Bar charts showing the proportion of each endothelial cells subtype totally and in each sample. (**d**) Violin plots representing the expression situation of common marker genes in different subtypes of endothelial cells. (**e**) Bubble chart showing five typical genes expressed in each subtype. (**f**) Heat map showing the top 10 marker genes of each subpopulation. (**g**) Pseudotemporal trajectory of all endothelial cells divided into five stages. (**h**) Pseudotemporal trajectory of five endothelial cells types. (**i**) Pseudotime trajectory showing the location of cells from each tissue during differentiation process. (**j**) The trajectory showing the distribution of four subpopulations in five samples. (**k**) Heat map for clustering the top genes that affected cell fate decisions

As the second most abundant cell after the epithelial cells, endothelial cells could be divided into four subpopulations, which were vascular endothelial cells (VECs), capillary endothelial cells (CapECs), lymphatic endothelial cells (LECs) and arterial endothelial cells (AECs) (Fig. 5a). All four subpopulations were detected in every sample (Fig. 5b). The proportion of each subtype was calculated in total and in each sample, except for that N1 did not contain LECs (Fig. 5c). In addition, we used a method to enable detailed classification of cell subsets based on the expression of typical marker genes in the subtypes of mononuclear phagocytes (Fig. 5d, e). The top ten expressed genes enriched our understanding of the various subpopulations (Fig. 5f). Pseudo-time trajectories divided all cells into five stages according to the key time nodes (Fig. 5 g). And we could notice that LECs were mainly clustered at the initiation stage of differentiation while other three clusters had distribution at all stages (Fig. 5 h). In order to clarify the heterogeneity between samples, we fit on the curve with a certain local cell as an independent unit and found that N5 was mainly concentrated at the beginning and the end (Fig. 5i). Also, the distribution of each sample on the proposed time trajectories showed significant individual differences (Fig. 5j). We also presented the changes of the expression of markers during the pseudotime developing process (Fig. 5k).

**Fig. 6:**
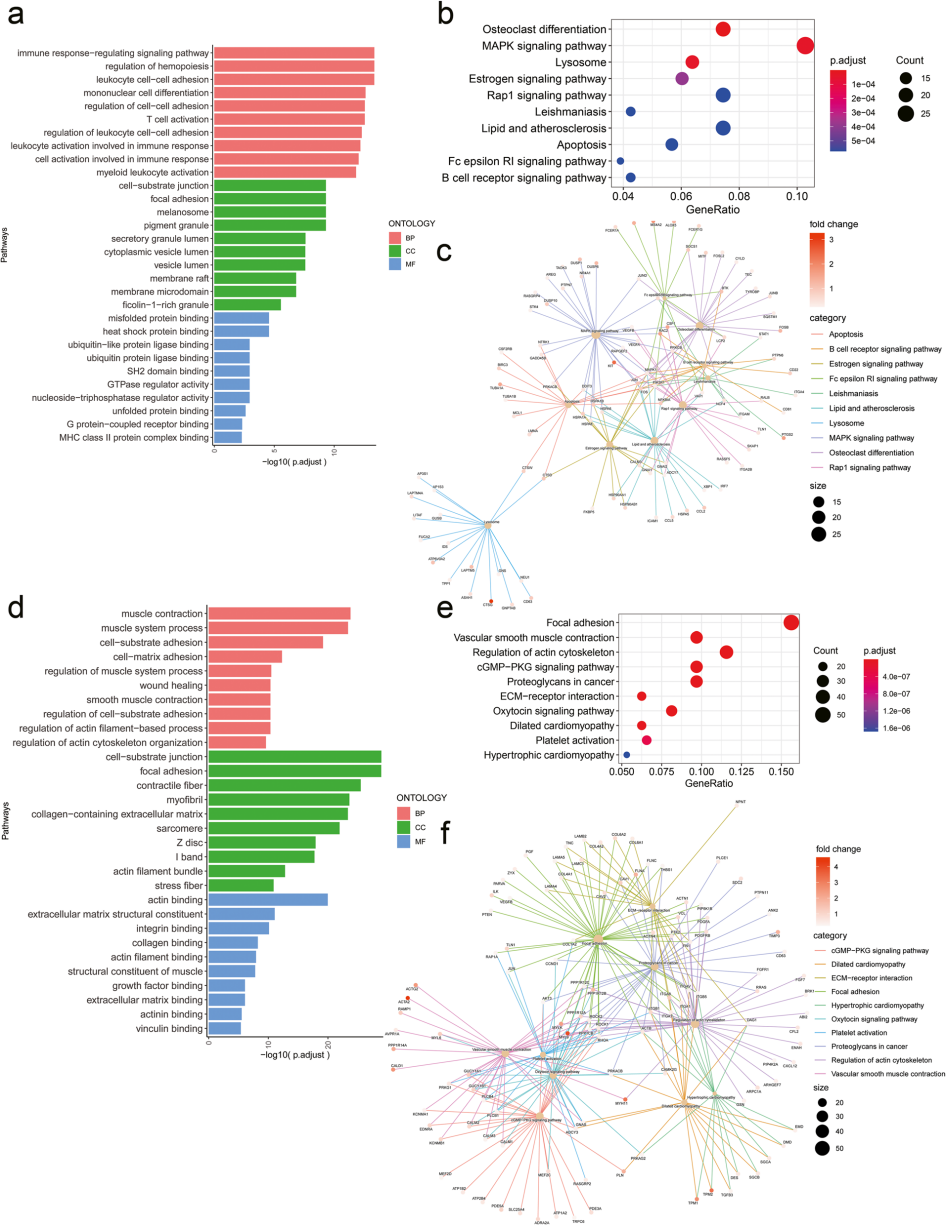
Functional enrichment analysis of mast cells and mural cells. (**a**) Bar chart showing the GO analysis of mast cells. (**b**) Bubble chart representing KEGG pathways associated with mast cells. (**c**) Mapplot showing the connection between enriched pathways of mast cells. (**d**) Bar chart showing the GO analysis of mural cells. (**e**) Bubble chart representing KEGG pathways associated with mural cells. (**f**) Mapplot showing the connection between enriched pathways of mural cells

Even though the proportion of mast cells and mural cells is small, we still believe that they play an important regulatory role in the microenvironment of normal adjacent tissues. We focused on their functions by GO analysis and KEGG analysis. The GO analysis of mast cells mainly fasten on the immunomodulating activity, as it is a type of immune cells (Fig. 6a). However, we noticed many essential pathways associated with mast cells, including MAPK signaling pathway, osteoclast differentiation, apoptosis and other ways (Fig. 6b). So we analysed the potential connection between the enriched pathways (Fig. 6c). The GO analysis of mural cells was mainly about extracellular matrix and muscle contraction (Fig. 6d). KEGG analysis was similar with GO results showing Focal adhesion, ECM-receptor interaction and many others (Fig. 6e). Similarly we analysed the potential connection between the enriched pathways (Fig. 6f).

The largest number of immune cells was the MPs at 3987 cells, more than 10% of the total number. In our analysis, MPs were classified into five subpopulations, which were monocytes, macrophages, mature dendritic cells, conventional type 1 dendritic cells (cDC1) and conventional types 2 dendritic cells (cDc2) (Fig. S2).

As an important component of immune system, T cells also require further assessment. In this study, a total of 884 T cells were noted, about 2.25% of the total number, which is not surprising as T cells play a significant role in cellular immunity under normal physiological conditions. In our analysis, T cells were also classified into five subpopulations, including CD4 + naive T cells, Treg, CD8 + Teff, proliferating T cells and innate lymphoid cells (ILCs) (Fig. S3).

**Fig. 7:**
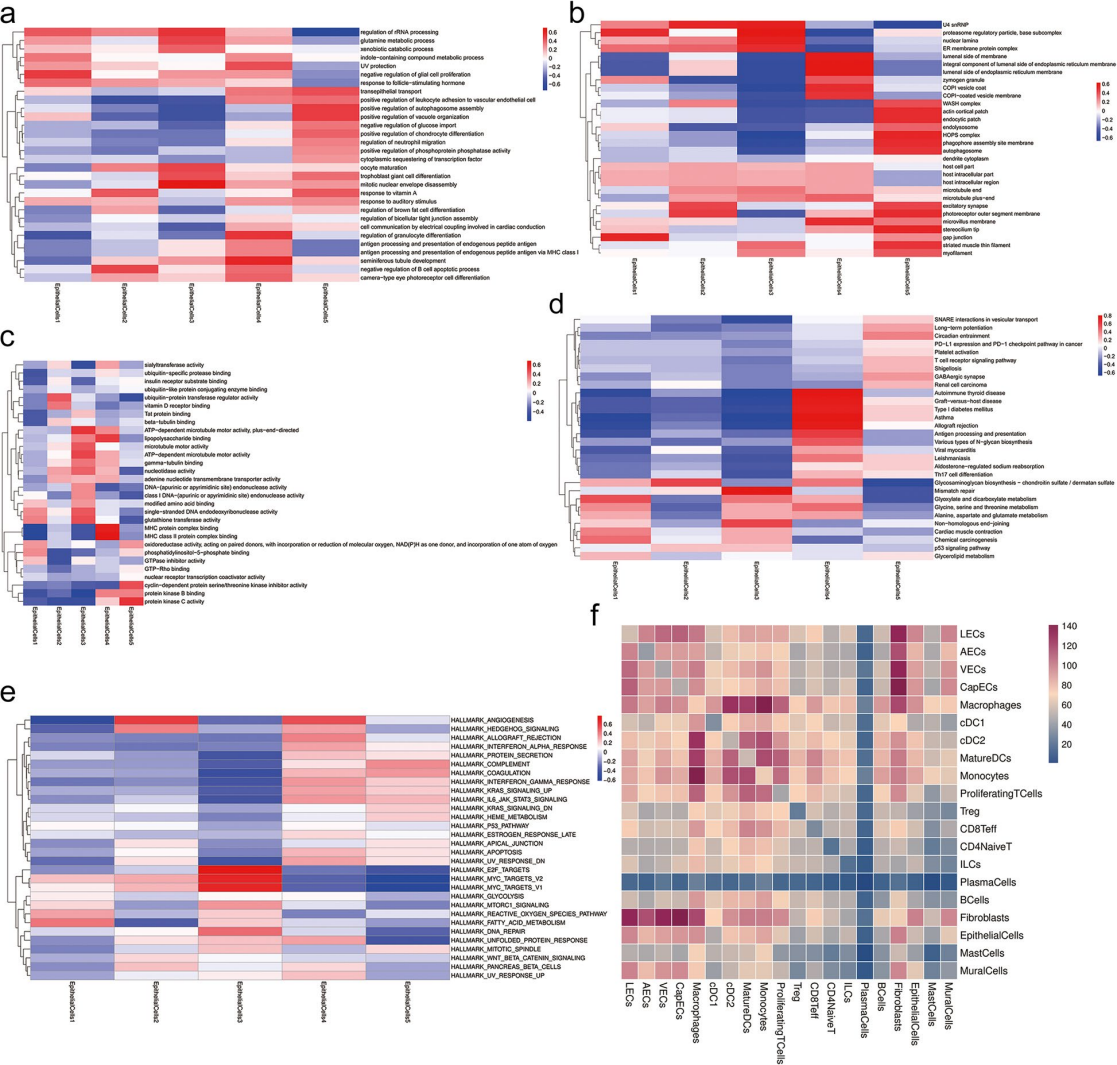
Functional subdivision of epithelial cells and interaction analysis between different cells. (**a**) Heatmap showing the key biological processes that different subtypes involved in. (**b**) Heatmap showing the cellular component which means the structural location where the gene product performs its function in each subtype. (**c**) Heatmap showing the major biological processes in which multiple molecules are involved in each subtype of epithelial cells. (**d**) Heatmap showing the signaling pathways involved in different kind of subpopulations. (**e**) Heatmap showing the top hallmarkers of 5 subtypes. (**f**) Heatmap showing the number of pairs of the interaction between each two cell types, the darker the color, the higher the number of pairs

As the identity and functions of the epithelial cell subtypes was unknown, we performed further functional (Fig. 7a-c) and pathway analyses (Fig. 7d) for each subtype. The tissues that removed from around the patients’ hypopharynx were normal paracancerous tissues. Our analysis of these tissues, their subtype cell clusters and genes enabled us to propose potential biomarkers (Fig. 7e) and will also enable further exploration of the interactions between different cell types (Fig. 7f).

## Discussion

Histologically normal tissue adjacent to the tumor (NAT) is commonly used as a control in cancer study. In our previous research, we used the five normal adjacent tissue samples as control to analyze the immune microenvironment in tumor tissues and metastatic lymphoid tissues cancer studies [27]. The study of NAT tissue has been first described as the “field cancerization” theory [47], then many studies suggested that the microenvironment surrounding the tumor is essential for understanding recurrence and in developing surgical strategies [30]. There were a large number of mutations and clonal expansions in adjacent normal epithelial tissues. Moreover, early clonal development and eventual cancer formation in paracancerous tissues have potentially different molecular mechanisms [14]. Also, there were large amount of chromosomal alterations [48], somatic mutations and clonal dynamics [49] in NATs. Using normal adjacent tissue as this control has many advantages, however, in comparing only tumor and NAT tissues, many potential cancer biomarker candidates may be missed and others spuriously implicated. Direct study of the specific cell populations in the adjacent tissues will help us to find the possible therapeutic target cells and targets in the adjacent tissues.

The purpose of our single-cell sequencing is not only to understand the classification of cell subtypes in normal adjacent to hypopharyngeal cancer tissues and the expression of heterogeneous genes connected with various cell–cell interactions, but also to provide database for researchers to refer to when they study the mechanisms related to hypopharyngeal cancer. From this point of view, we need to pay attention to some targets and indications related to cancer initiation, development and prognosis in our classification and research results.

As mentioned above, epithelial cells make up the largest proportion of all cells, so its role in normal tissues deserved discussion. Based on the top five expressed genes in five different subpopulations, we tend to define genes that are highly expressed in all five subpopulations detected as marker genes for epithelial cells, namely CSTB, KRT13, and KRT4. The large number of cell subsets were basically in the terminal stage of cell development and differentiation in quasi-chronological analysis, and cells in the initial stage with strong ancestry accounted for less in the five samples. Besides, the subset epithelial cells 5 in the initial stage was only detected in two tissue samples, which led us to consider whether there was a correlation between the heterogeneity of epithelial cell subsets and their sensitivity to cancer-related factors between different patients.

When we consider the role of epithelial cells in cancer, the first thing that usually comes to mind is epithelial-mesenchymal transformation (EMT) [50]. Under normal conditions, epithelial cells play a protective and resorptive role in tissues, but in the process of the development of various malignant tumors, with gene mutations [51] or under the induction of inflammatory factors [52], tumor cells can obtain invasive characteristics through EMT, infiltrate into the surrounding stroma, and form a microenvironment that promotes tumor growth and metastasis [52]. Therefore, we hope that subsequent innovative studies can find some predictive genes related to the risk factor of epithelial-mesenchymal transformation of tumor tissue by comparing it with the normal marker in our database, so as to target it to play a blocking role.

As the main component of non-immune cells in the microenvironment, cancer-associated fibroblasts (CAFs) plays an essential role in the development of carcinoma, the signal transduction between different subtypes of CAFs and immune cells can promote tumor development, invasion and metastasis [54]. The genes that highly expressed in the two subtypes can affect HNSCC patients’ survival time. RGS5 highly expressed fibroblasts is clustered as vascular CAFs [55], and the fibroblasts in our study is associated with TNF, EGF and chemokine related functions. In the two subtypes of fibroblasts, APOD gene presents a high expression state. As a member of the lipoprotein family, APOD is mainly produced by the brain and testes. Lipoprotein is involved in the lipid transport metabolism process [56], and also plays a vital role in the aging process of various organs [57], so the relationship between APOD and fibroblasts in our sequencing results needs to be further studied. The most noteworthy is the significant difference in the proportions of the two subgroups among the five patients. We will follow up the recovery and survival of the patients after cancer surgery to find out the role of the two subgroups in the severity of the disease.

Tumor endothelial cells (TECs) is associated with angiogenesis during tumor development, its production and tumor progression promote each other [58]. Apart from this, it is also involved in other biological processes such as immune regulation [59] and extracellular matrix composition [60]. Antiangiogenic therapies provide clinical and survival benefits in patients with many types of cancer by inhibiting angiogenesis and endothelial cells. The heterogeneity of tumor endothelial cells makes anti-angiogenic therapies have significant differences in efficacy and drug resistance [61]. As angiogenesis is one of the hallmarks of cancer, anti-angiogenic therapy (AAT) is widely used in a variety of cancers. A research suggested a pan-cancer mechanism of pro-inflammatory signals from the tumor stimulates an inflammatory response in the adjacent endothelium [11], which means not only normal adjacent tissues can simulate the changes in the early stages of cancer, but only tumors can affect surrounding endothelial cells.

Mast cells are granulocytes that mediate host defense and maintenance of homeostasis by swiftly degranulating histamines, cytokines, and chemokines. They are well known for their role in allergies and autoimmunity, but they can also infiltrate tumors. Mast cells exert both pro- and anti-tumorigenic activities depending on the microenvironmental stimuli. They can directly target tumor cells, but they mainly regulate the recruitment and activity of other immune populations and the endothelium [62]. In our study, mast cells also related with apoptosis and other non-immunal pathways, which attracted our attention.

Mural cells are microcirculating vascular smooth muscle cells (vSMC) and pericytes. Both types are in close contact with the endothelial cells lining the capillaries and are important for vessel development and stability. Parietal cells are involved in the formation of normal vasculature and respond to factors such as platelet-derived growth factor B (PDGFB) and vascular endothelial growth factor (VEGF) [63]. Mast cells and endothelial cells are the main members of the vasculature in tumors, which is a key component of the tumor microenvironment with critical roles in regulating metastatic seeding and progression [64]. In our results, we found mural cells played important roles in extracellular environment, this may promote the transformation from adjacent status to cancer status and meanwhile affect the malignant degree of carcinoma.

As the largest proportion of immune-related cells in our sequencing results, monocytes are a subpopulation of white blood cells that play a key role in maintaining homeostasis, pathogen recognition and clearance, as well as inflammation. We found that in the five samples, macrophages invariably accounted for the largest cell population. As an ‘undercover agent’ in the immune system, macrophages can undergo different activation in different environments, thus forming subsets with different molecular and functional characteristics, and are key effector cells of innate immunity with powerful phagocytosis. Activated macrophages mainly include M1 macrophages and M2 macrophages, M1 macrophages can kill tumor cells and resist pathogen invasion, and M2 macrophages, which account for the vast majority of tumor tissues, mainly play a role in promoting tumor growth, invasion and metastasis [65]. Therefore, macrophages can also be used as a follow-up research focus.

Except for macrophage, other cell subtypes, cDC1 and cDC2 also attracted our attention, with studies showing that conventional type 1 dendritic cells (cDC1) are thought to perform antigen cross-presentation, which is required to activate CD8 + T cells, while cDC2 [66] is dedicated to activating CD4 + T cells. CD4 + T cells are also thought to help CD8 + T cell responses through a variety of mechanisms [67]. The expression of cDC1 and cDC2 is also consistent with the detection results of CD8 Teff and CD4 Naive T cells in the T cell subpopulation of our sequence, demonstrating the accuracy of our sequencing results.

T cells, as the main force of cellular immunity in normal tissues, were also significantly detected in the sequencing results. Unexpectedly, proliferating T cells still account for a large proportion, and even account for more than half of the samples in one clinical sample. We think these results prove that as a common pathway for respiratory digestion, hypopharynx adapts to stronger immunity in the face of more complex bacterial and viral environments.

Considering the data types of various cells, finally, we still analyzed the interaction mode between different subtypes of epithelial cells, the place of action and enrichment pathways of metabolites, and markers with follow-up exploration significance. Through the analysis of correlations between different cell subtypes, we hope to provide some new combinations of cells with interconnected influences for subsequent cellular microenvironment analysis and interaction studies.

## Conclusion

In conclusion, we have provided a transcriptome profile of normal adjacent tissues in hypopharyngeal carcinoma. The data we report here extend our current understanding of the tumor-related microenvironment in normal tissues at early stage of the disease at the molecular level, investigate hypopharyngeal cell biology and the relationship between cell types and disease, and follow up with further taxonomic studies of nonimmune components in the tissue microenvironment.

### Data records

All hypopharyngeal sequence data have been uploaded to NCBI GEO database. The raw data for the BAM file has been archived in the NCBI Sequence Read Archive (SRA) and can be accessed under project accession number GSE206038.

## Fundings

This work was supported by the National Natural Science Foundation of China (No. 82071918, No. 82203770).

### Supplementary Information

**Supplementary Material 1.**
