# Supplementary material for: Single-cell transcriptomic landscape and the microenvironment of normal adjacent tissues in hypopharyngeal carcinoma
Source: BMC Genomics. 2024 May 17;25:489. doi: 10.1186/s12864-024-10321-2 (PMC11100249; doi:10.1186/s12864-024-10321-2)

Supplementary Figure legends


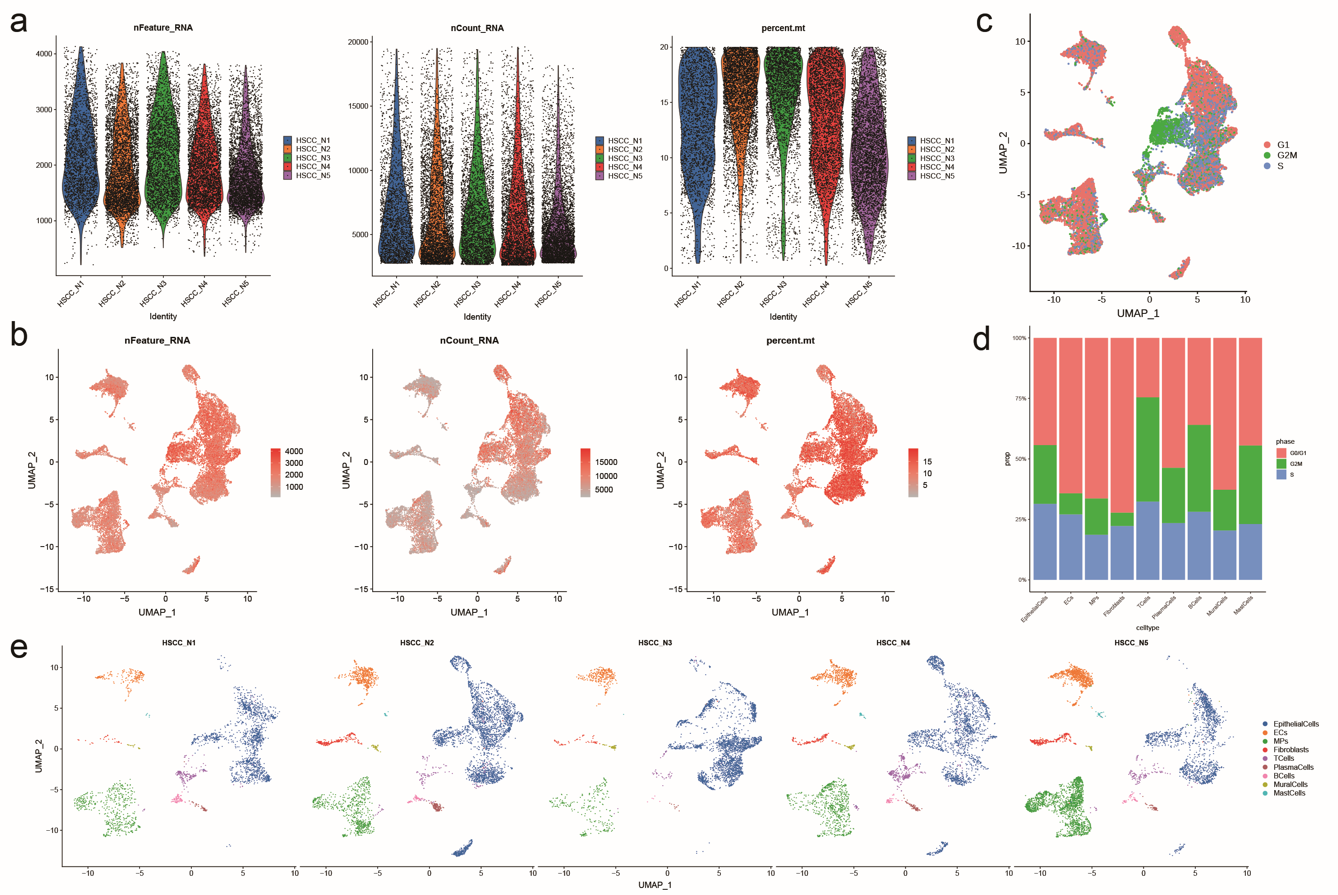


Supplementary Figure S1 Quality control (QC) of human hypopharynx single cell data.

(a) Scatterplot illustrating the number of genes, unique molecular identifiers (UMIs) and the percentage of mitochondrial genes in each cell of five hypopharynx samples. (b) The number of genes, unique molecular identifiers (UMIs) and the percentage of mitochondrial genes of all samples are showed in the form of UMAP plot. (c) UMAP plot showing the cell cycle status of each cell. (d) Bar chart showing the proportion of cells in different cycle status of each subpopulation. (e) We detected the batch effect between five different hypopharynx samples.


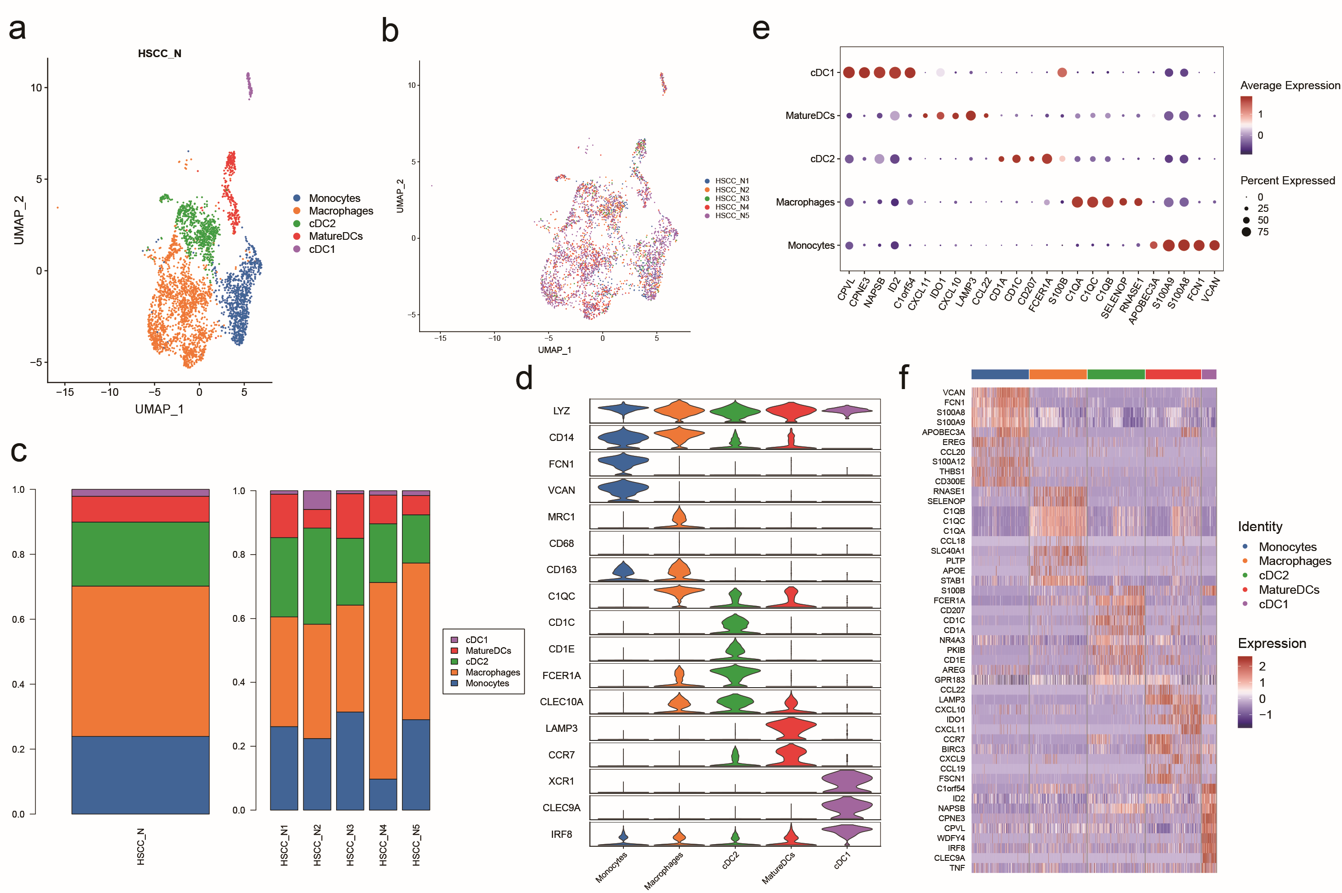


Supplementary Figure S2 Subpopulations and markers of mononuclear phagocytes.

(a) Uniform manifold approximation and projection (UMAP) plot showing the sub classification of mononuclear phagocytes. (b) UMAP plot of five subtypes colored by samples. (c) Bar charts showing the proportion of each mononuclear phagocytes subtype totally and in each sample. (d) Violin plots representing the expression situation of common marker genes in different subtypes of mononuclear phagocytes. (e) Bubble chart showing 5 typical genes expressed in each subtype. (f) Heat map showing the top 10 marker genes of each subpopulation.


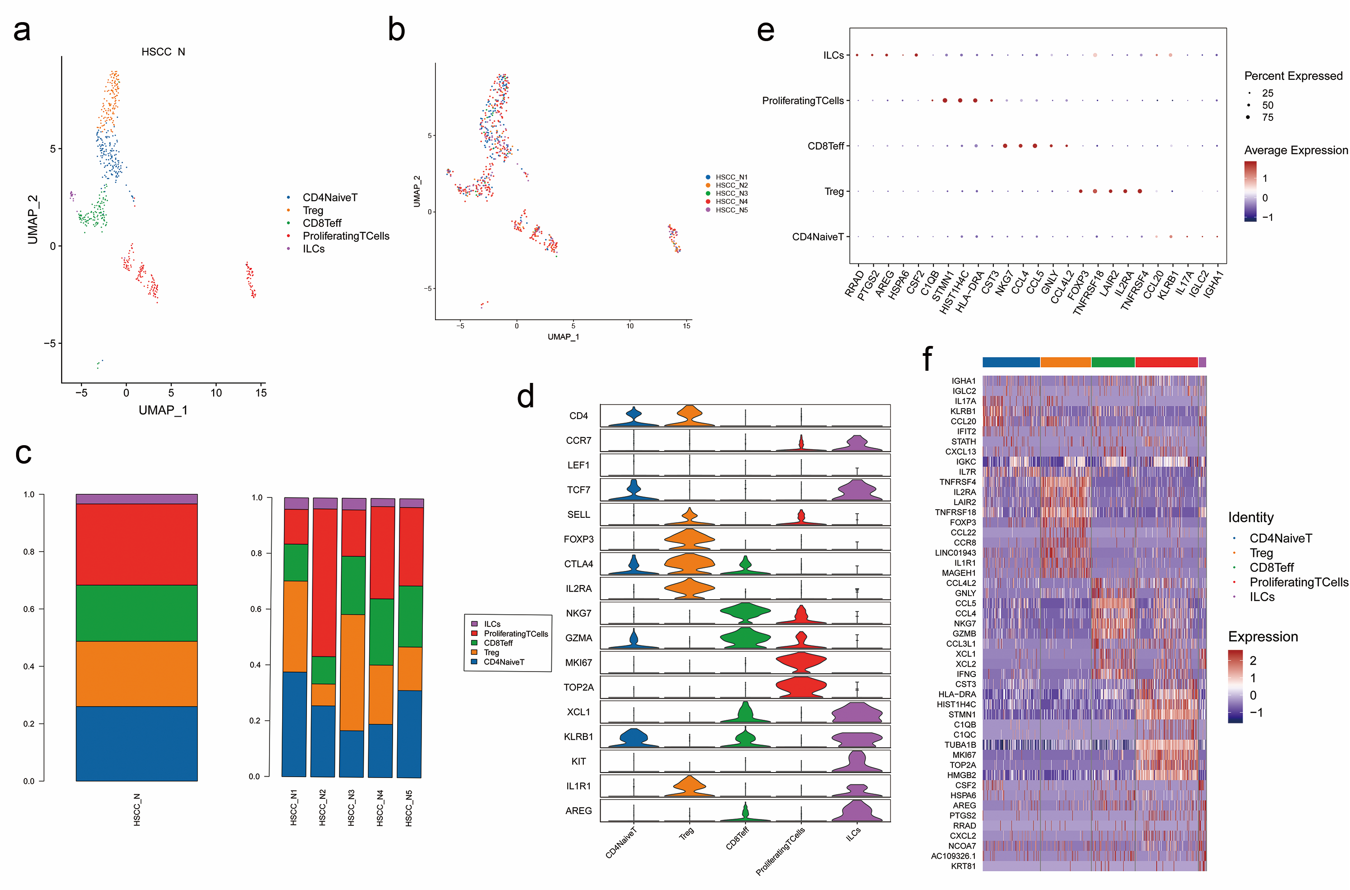


Supplementary Figure S3 Subpopulations and markers of T cells.

(a) Uniform manifold approximation and projection (UMAP) plot showing the sub classification of T cells. (b) UMAP plot of five subtypes colored by samples. (c) Bar charts showing the proportion of each T cells subtype totally and in each sample. (d) Violin plots representing the expression situation of common marker genes in different subtypes of T cells. (e) Bubble chart showing 5 typical genes expressed in each subtype. (f) Heat map showing the top 10 marker genes of each subpopulation.

Supplementary Table legends

Supplementary Table 1 Information of five different patients.


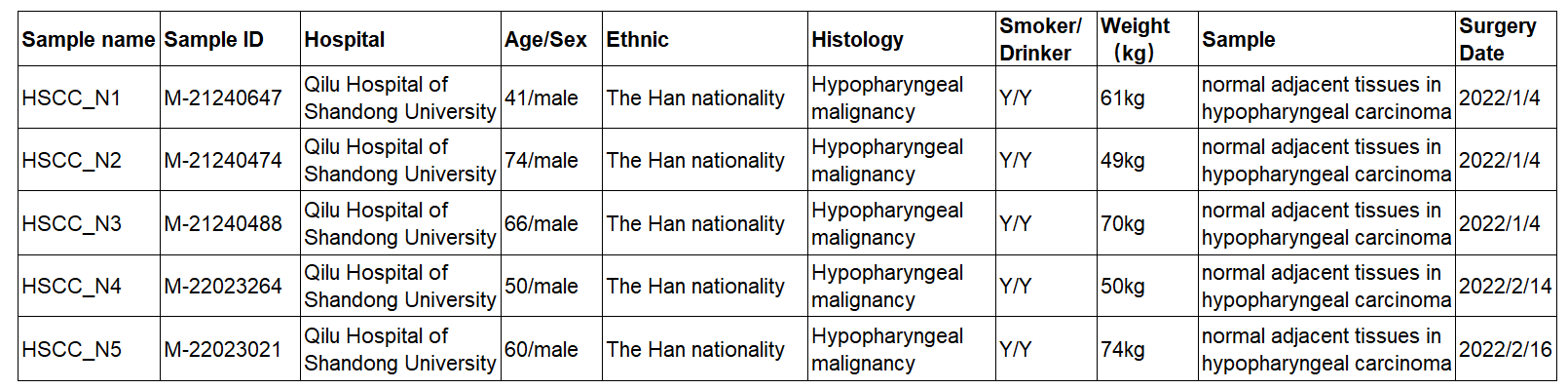


Supplementary Table 2 Basic information and dissociation results of five tissue samples


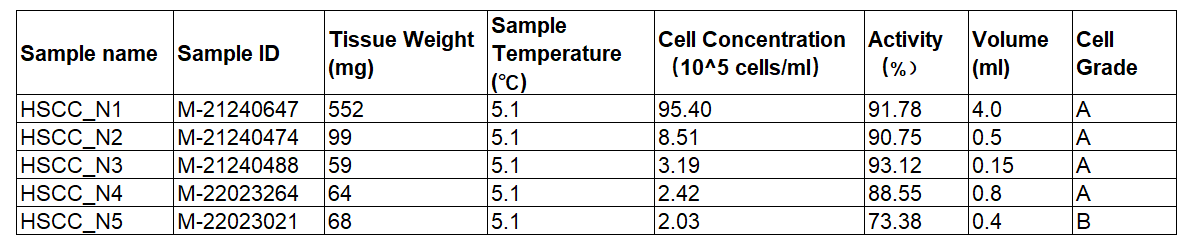

Supplement: Supplementary file 1 — Supplementary Material 1. [file 12864_2024_10321_MOESM1_ESM.zip › Supplemental material/Supplemental materials.docx]
