# Supplementary figures and images for: Single-cell transcriptomic landscape and the microenvironment of normal adjacent tissues in hypopharyngeal carcinoma
Source: BMC Genomics. 2024 May 17;25:489. doi: 10.1186/s12864-024-10321-2 (PMC11100249; doi:10.1186/s12864-024-10321-2)

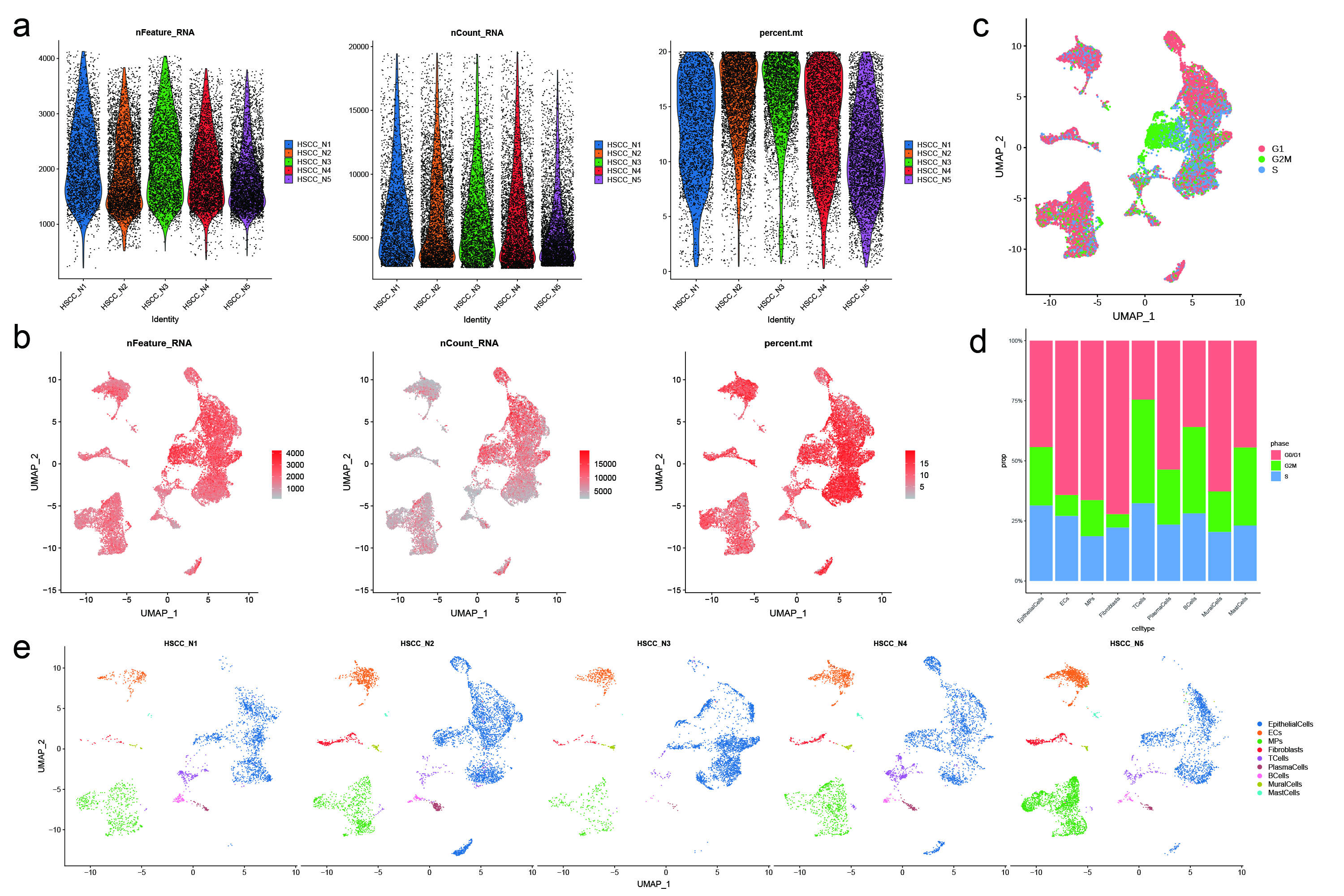

Supplement: Supplementary file 1 — Supplementary Material 1. [file 12864_2024_10321_MOESM1_ESM.zip › Supplemental material/Supplementary Figure S1.tif]

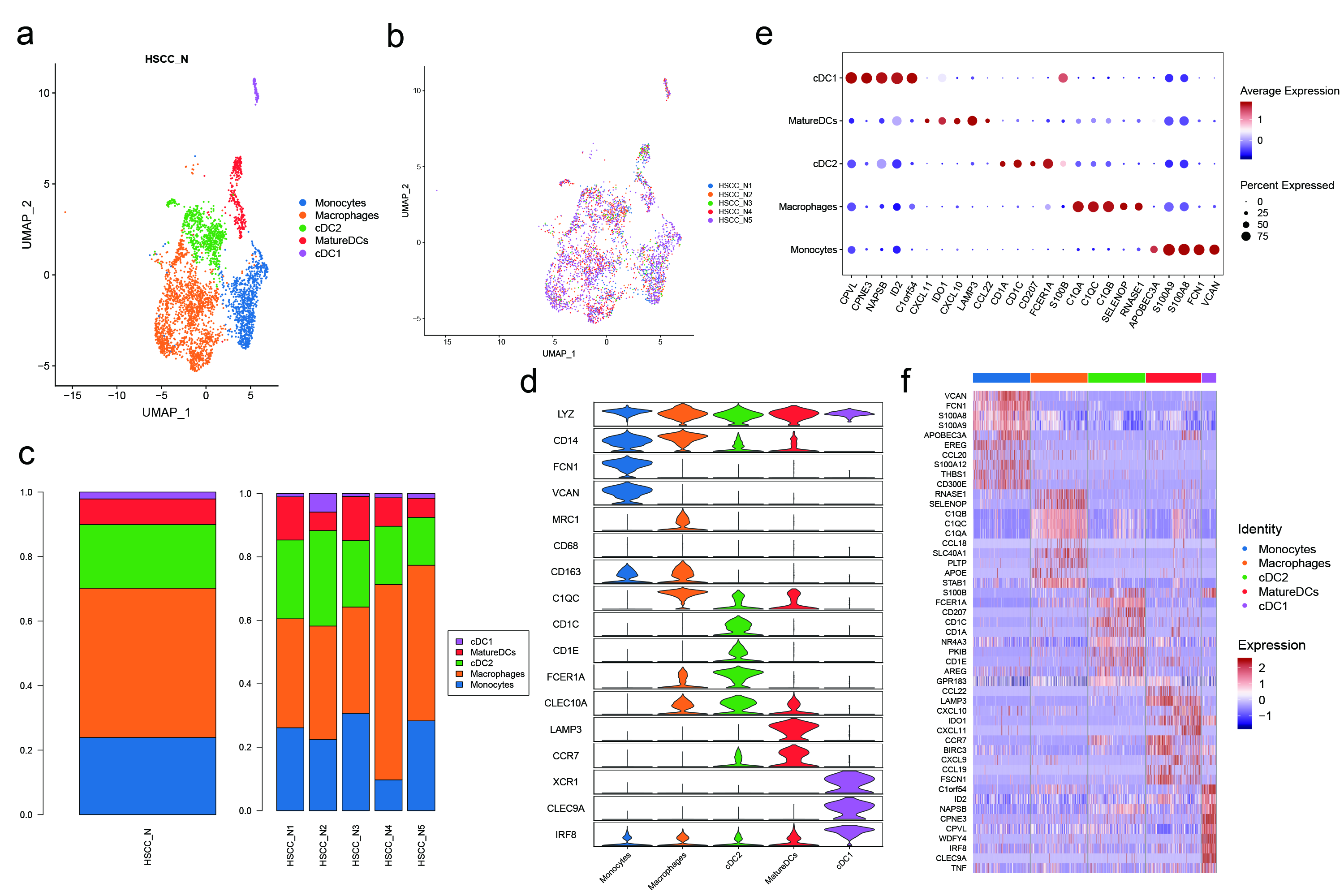

Supplement: Supplementary file 1 — Supplementary Material 1. [file 12864_2024_10321_MOESM1_ESM.zip › Supplemental material/Supplementary Figure S2.tif]

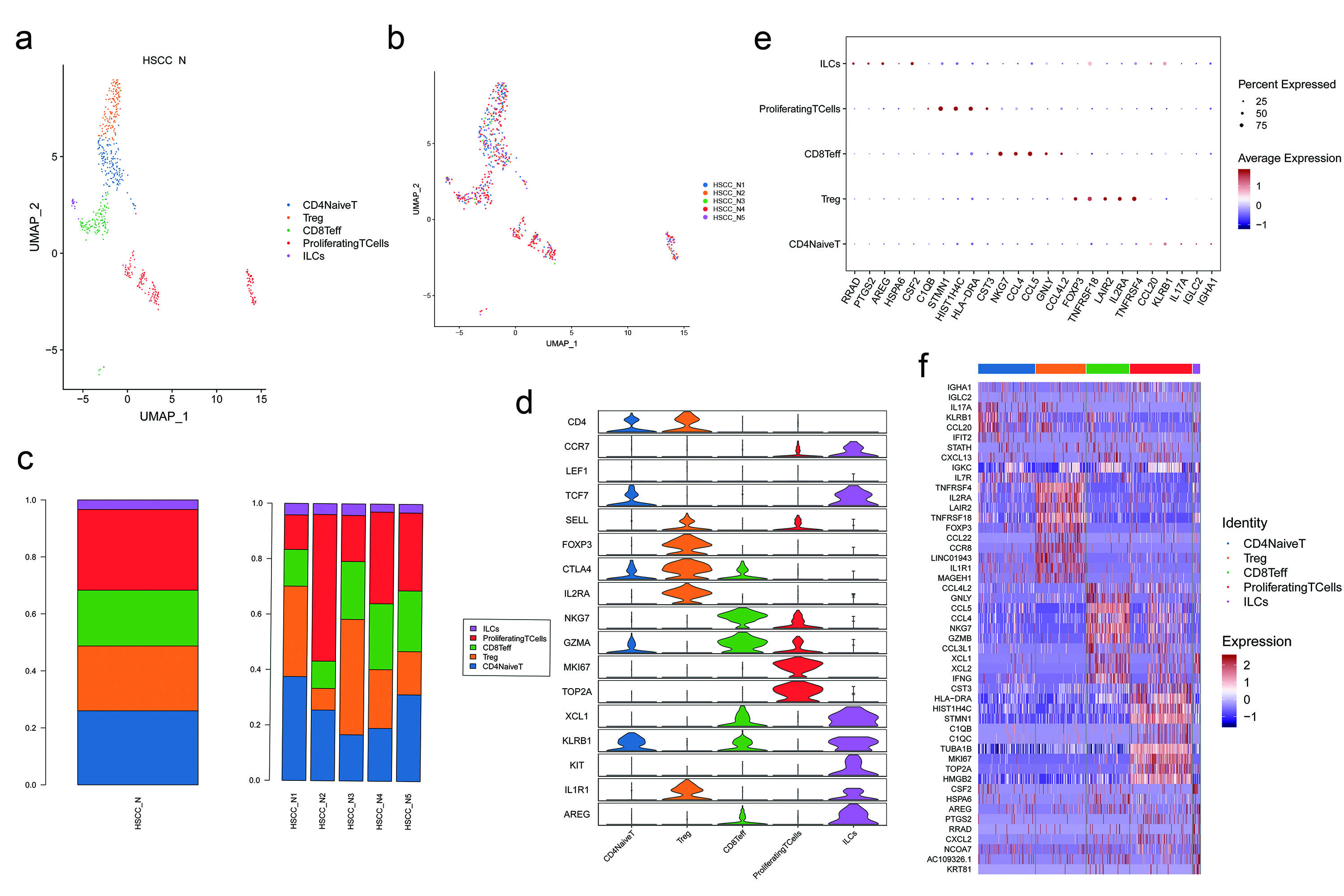

Supplement: Supplementary file 1 — Supplementary Material 1. [file 12864_2024_10321_MOESM1_ESM.zip › Supplemental material/Supplementary Figure S3.tif]
